# The Effectiveness of Interventions for Developmental Dyslexia: Rhythmic Reading Training Compared With Hemisphere-Specific Stimulation and Action Video Games

**DOI:** 10.3389/fpsyg.2020.01158

**Published:** 2020-06-03

**Authors:** Alice Cancer, Silvia Bonacina, Alessandro Antonietti, Antonio Salandi, Massimo Molteni, Maria Luisa Lorusso

**Affiliations:** ^1^Department of Psychology, Catholic University of the Sacred Heart, Milan, Italy; ^2^Auditory Neuroscience Laboratory, Northwestern University, Evanston, IL, United States; ^3^Department of Communication Sciences and Disorders, Northwestern University, Evanston, IL, United States; ^4^Unit of Child Psychopathology, Scientific Institute IRCCS ‘Eugenio Medea’, Bosisio Parini, Italy

**Keywords:** reading, developmental dyslexia, music, rhythm, action video games, hemisphere-specific stimulation

## Abstract

Developmental dyslexia is a very common learning disorder causing an impairment in reading ability. Although the core deficit underlying dyslexia is still under debate, significant agreement is reached in the literature that dyslexia is related to a specific deficit in the phonological representation of speech sounds. Many studies also reported an association between reading skills and music. These findings suggest that interventions aimed at enhancing basic auditory skills of children with DD may impact reading abilities. However, music education alone failed to produce improvements in reading skills comparable to those resulting from traditional intervention methods for DD. Therefore, a computer-assisted intervention method, called Rhythmic Reading Training (RRT), which combines sublexical reading exercises with rhythm processing, was implemented. The purpose of the present study was to compare the effectiveness of RRT and that of an intervention resulting from the combination of two yet validated treatments for dyslexia, namely, Bakker’s Visual Hemisphere-Specific Stimulation (VHSS) and the Action Video Game Training (AVG). Both interventions, administered for 13 h over 9 days, significantly improved reading speed and accuracy of a group of Italian students with dyslexia aged 8–14. However, each intervention program produced improvements that were more evident in specific reading parameters: RRT was more effective for improvement of pseudoword reading speed, whereas VHSS + AVG was more effective in increasing general reading accuracy. Such different effects were found to be associated with different cognitive mechanisms, namely, phonological awareness for RRT and rapid automatized naming for VHSS + AVG, thus explaining the specific contribution of each training approach. Clinical Trial registration: ClinicalTrials.gov NCT02791841.

## Introduction

Developmental dyslexia (DD) is one of the most common learning disabilities. It is unanimously considered to be a neurobiological disorder – even though its brain bases are still under debate (Norton et al., 2015) – characterized by both deficiencies in accurately and fluently recognizing words, poor spelling, and deficits in decoding, despite there being no evidence of an association to any specific sensory, neurological, or intellectual deficiencies (Lyon et al., 2003).

Among the several etiological hypotheses proposed, significant agreement is reached in the literature that DD is related to a specific deficit in the phonological representation of speech sounds (Goswami et al., 2002; Ramus, 2004; Ramus et al., 2013; Castles and Friedmann, 2014). At the same time, there are also studies focusing on different functions, such as long-term and short-term verbal memory, working memory, visual perception, auditory perception, automatization and learning functions, and spatial attention (e.g., Heim and Grande, 2012). In fact, many researchers (e.g., Pennington, 2006) are converging on the idea that several causes and factors interact to contribute to the emergence of reading disorders.

This lively search for DD brain basis is fostering a new wave of innovative treatments aiming at enhancing reading skills in an indirect way, by training the cognitive and perceptual skills potentially involved in the reading process (e.g., Thomson et al., 2012; Bhide et al., 2013; Flaugnacco et al., 2015; Cancer and Antonietti, 2018; Frey et al., 2019; Pecini et al., 2019). However, there remains a strong need to identify among old and new interventions the ones that are, first of all, effective in improving reading skills (Lorusso et al., 2006) and, secondly, use resources efficiently (Franceschini et al., 2013). In addition, interventions should be challenging for and appealing to children, this being one of the major requests by those who carry out treatments.

Taking up the challenge to come up with an intervention that meets all of these three goals, herein we will discuss the effectiveness of a new computer-assisted training called Rhythmic Reading Training (RRT). The benefits of the latter in improving reading skills in children with DD have already been shown when compared to a control group (Bonacina et al., 2015). The present study will extend these findings by a comparison with another remediation package for poor readers recently developed in the Italian clinical practice.

The theoretical basis of RRT lies in the fact that children with DD fail to detect supra-segmental cues of speech, such as rhythm, pitch, and stress (Goswami et al., 2002; Huss et al., 2011), even before failing to manipulate segmental cues, the latter being a crucial skill to learn the grapheme-phoneme correspondence (Richardson et al., 2004). More precisely, according to the temporal sample framework hypothesis, this impairment in capturing stress and rhythmic patterns reflects a more general temporal encoding deficit in individuals with DD, which in particular seems to affect the slower temporal rate in speech processing and tracking of the amplitude envelope and, indeed, it has been shown to have an impact on phonological development (Goswami, 2011).

Following Goswami’s suggestions for the ideal DD intervention (Goswami, 2011), RRT is built integrating musical, rhythmical, other supra-segmental, and segmental elements. The potential importance of music in training for DD is in line with the emerging literature investigating the power of music to induce plastic changes and perceptual enhancements within the neural system crucial for reading (Kraus et al., 2014a, b). However, music education programs alone seem to be less effective in improving reading skills than a traditional language-based intervention for DD and should not completely replace traditional methods (Kraus and Chandrasekaran, 2010). Studies to date have shown that music trainings by themselves enhance phonological awareness skills, but may require more intensive training to produce stronger measurable improvements on reading fluency (Overy, 2003; Cogo-Moreira et al., 2013; Gordon et al., 2015; Habib et al., 2016). RRT’s exercises, in which musical and linguistic dimensions are merged, are therefore designed to provide a stronger unified approach. In particular, RRT integrates the traditional sublexical treatment remediation approach, which has been proven to be one of the two most effective treatments for DD in the Italian background (Tressoldi et al., 2003), with a rhythmic component. In this way, the rhythmic accompaniment should provide readers an anchor to help them organize temporal cues of speech sounds so as to facilitate the automatization of the grapheme-phoneme correspondence in reading. More concretely, RRT consists of a child-friendly computerized reading program which was designed with an interactive and pleasant interface to engage and sustain the children’s attention during the treatment sessions. The interested reader can find more details in Bonacina et al. (2015) and Cancer et al. (2016).

Taking all RRT features into account, we decided to compare the impact of RRT with a remediation intervention which is made up by combining two training programs: (a) a deep-rooted treatment for DD devised after Bakker’s Balance Model and known as Visual Hemisphere-Specific Stimulation (VHSS), which is one of the most effective treatment for DD in the Italian background, according to the results of a large multicenter study comparing various types of treatments used in Italy by Tressoldi et al. (2003) and (b) a recently proposed intervention which consists of playing action-videogames by the Nintendo Wii technology (Franceschini et al., 2013). Both these treatments in isolation have already documented their effectiveness in improving reading skills in Italian children with DD (for a recent systematic review, see Peters et al., 2019). In particular, (a) the VHSS treatment promoted a significant and stable enhancement in both reading fluency and accuracy (Lorusso et al., 2006, 2011; Peters et al., 2019), and (b) the action-videogames improved children’s reading speed without any cost in accuracy (Franceschini et al., 2013; Gori et al., 2016; Peters et al., 2019). The comparison of results of an intervention based on the combination of these two treatments, one of long-lasting evidence of effectiveness and another clearly going beyond the traditional purely linguistic component, seemed both a reasonable and challenging choice for RRT, which aims at reaching the same two objectives thanks to only one (that is, resulting from the combination of two different methods in one single module) homogeneous training program.

Both interventions are characterized by strengths and weaknesses in their application. On the one hand, RRT appears to have some compelling and practical advantages. Firstly, it is implemented using an accessible and usable technology, i.e., a software that can be run on any computer, and which requires only a short training for the clinician. Secondly, whereas the VHSS-AVG intervention requires the child to take part in two separate sessions, only one of which involves the videogames, in RRT the musical and linguistic components are always merged and proposed via the interactive software. On the other hand, the presence of a videogame module with no obvious link to reading remediation may be an effective way to lower pressure and anxiety and increase motivation and compliance in more frustration-prone individuals.

The present study should provide valuable information about the specific effects of the two programs on both reading performance and reading-related functions. Correlations between reading improvement following treatment and gains in other cognitive functions had been computed in previous studies comparing the effects of various intervention programs (Lorusso et al., 2006, 2011). Since no such analysis has ever been conducted on the effects of the combined VHSS-AVG treatment, nor of the RRT treatment, an exploratory analysis of the correlation between primary and secondary outcome measures appeared to give interesting information on the mechanisms involved in improvement.

## Materials and Methods

### Participants

Twenty-four Italian students aged between 8 and 14 years (mean age = 9.79 years, *SD* = 1.64) with a diagnosis of DD were involved in the study. Regarding the sample size, previous studies (Lorusso et al., 2006, 2011; Bonacina et al., 2015) showed that expected improvements in reading ability (pre/post-test gains in *z*-scores for text reading speed and accuracy) were about 1.2 *z*-scores (*SD* ≈ 1). Under these conditions, statistical power against the hypothesis of a null gain with a sample size of 12 would be 97% two-tailed, 98.5% one-tailed (alpha error = 5%). Therefore, we assumed that a sample size of 12 for each group is appropriate for the present research.

Participants were recruited among patients of the neuropsychiatry unit of IRCCS ‘Eugenio Medea’ in Bosisio Parini, Northern Italy, in January 2016. The parents of the eligible participants were contacted by the researchers. The purpose and procedures of the study were explained in detail to both children and their parents. Written parental informed consent of those who agreed to take part in the study was obtained before the beginning of the treatment.

All the participants had to fulfill the following inclusion criteria: (a) having been previously diagnosed with DD (ICD-10 code: F81.0) on the basis of standard inclusion and exclusion criteria (ICD-10: World Health Organization, 1992) and of the diagnosis procedure followed in the Italian practice; (b) absence of comorbidity with other neuropsychiatric or psychopathological conditions (whereas comorbidity with other learning disabilities were allowed); (c) not having been involved in previous reading intervention programs.

The study was approved by the Psychology Research Ethics Committee of the Catholic University of the Sacred Heart, Milan, Italy on the 22nd December 2015 (Clinical Trial ID: NCT02791841; the trial registration was delayed for administrative reasons). The entire research process was conducted according to standards of the Helsinki Declaration.

### Interventions

Two subgroups of the same size were pseudo-randomly assigned to one of two experimental conditions so as to be matched for gender, age, IQ, dyslexia subtype – classified according to the Balance Model (Bakker and Licht, 1986) – and level of reading impairment (see Table 1): (1) RRT (Bonacina et al., 2015; Cancer et al., 2016) (*N* = 12) or (2) an intervention resulting from the combination of two previously validated treatments for DD: visual hemispheric-specific stimulation (VHSS) (Bakker and Vinke, 1985; Lorusso et al., 2006, 2011), and Action Video Game training (AVG) (Franceschini et al., 2013, 2015) (*N* = 12).

**TABLE 1:** Participants’ characteristics.

	VHSS-AVG *N* = 12	RRT *N* = 12	Group comparison	
	Mean (*SD*)	Mean (*SD*)	Mann–Whitney test: U, *p*	χ ^2^, *p*
Age	9.67 (1.37)	9.92 (1.93)	72.00, 1.00	–
Male	7	7	–	0.00, 1.00
Female	5	5	–	
FSIQ^a^	94.08 (7.30)	99.45 (9.39)	47.00, 0.24	–
VIQ^a^	87.78 (7.50)	93.11 (11.63)	30.50, 0.38	–
PIQ^a^	98.67 (9.84)	103.33 (6.96)	28.50, 0.29	–
L-types^b^	1	2	–	2.75, 0.25
P-types^b^	7	3	–	
M-types^b^	4	7	–	
Text reading Speed^c^	−1.68(2.14)	−1.38(1.18)	61.00, 0.52	–
Text reading Accuracy^c^	−3.10(3.09)	−2.88(1.93)	65.00, 0.69	–
Word reading Sp.^c^	−3.19(2.91)	−3.65(4.73)	70.00, 0.91	–
Word reading Acc.^c^	−2.97(3.37)	−2.23(2.70)	64,00, 0.64	–
Pseudo-word reading Sp.^c^	−2.45(2.26)	−2.49(1.54)	72.00, 1.00	–
Pseudo-word reading Acc.c	−1.95(1.54)	−1.79(1.65)	71.00, 0.95	–
Phonemic Blending^d^	4,25(4.05)	4.58 (3.80)	65.00, 0.68	–
Phonemic Elision^d^	1.83 (1.90)	2.08 (2.11)	65.5, 0.70	–
RAN Speed^e^	−1.66(1.01)	−2.51(1.26)	47.5, 0.16	–
RAN Accuracy^e^	−1.85(1.65)	−0.96(2.07)	51.5, 0.23	–

*^*a*^Composite score derived from the Wechsler Intelligence Scale for Children Third Edition (WISC-III): Verbal IQ (VIQ), Performance IQ (PIQ), and Full Scale IQ (FSIQ). ^*b*^Types of dyslexic readers according to the Balance model. ^*c*^Scores are expressed as *z*-scores in the text, word and pseudo-word (from DDE-2 battery) reading tasks (positive *z*-scores represent above-average performance, negative *z*-scores below-average performance). ^*d*^Raw scores (n. of errors) in phonological tasks. ^*e*^Scores are expressed as *z*-scores in the Rapid Automatized Naming (RAN) task.*

#### Rhythmic Reading Training (RRT)

RRT is a child-friendly computerized reading training program designed for Italian students with DD aged 8–14 years. The main feature of this intervention is the integration of a traditional remediation approach (sublexical treatment) with rhythm processing. Accordingly, all reading exercises are characterized by a rhythmic accompaniment with gradually increasing speed. The acoustic stimulation used in RRT consists in an isochronous drumbeats rhythmic pattern. The beats feature alternating pulse intensity (i.e., weak and strong), in a metronome-fashion. The training program includes three categories of exercises, each aimed at training a specific reading ability: “Syllables,” “Merging,” and “Words and Pseudo-words.” Children are taught to read aloud the verbal stimuli (i.e., syllables, words, pseudo-words, phrases) presented on the screen in synchrony with the acoustic rhythm. In all exercises, the acoustic rhythmic pattern matches the metrical structure of language, so that when syllabic units constituting words are presented separately and sequentially, a stronger rhythmic beat stresses the accented syllable. During the first presentation of each exercise, the stimulus that has to be read is highlighted by a visual cue synchronized with rhythm, so to make it easier for children to read the verbal material at a specific pace. The software allows the trainer to modulate the rhythm pace adaptively, by setting faster rates as the child gained competence. The starting point is set according to the child’s reading speed at baseline, as measured in pre-training assessment. The easy-to-use interface and the intuitive settings of the software makes it very clear and easy to be managed by any trainer. RRT was proven to be effective in improving reading speed and reading accuracy of Italian children with DD, compared to spontaneous development. A test-training-retest study showed the efficacy of RRT intervention on reading abilities of 14 junior high school students with DD, compared to a matched control group that received no intervention (Bonacina et al., 2015). Other test-training-retest small scale studies supported RRT efficacy in primary and junior high-school Italian students with DD, under different conditions (Cancer and Antonietti, 2017; Cancer et al., 2019).

#### VHSS-AVG Training

This training combines VHSS according to Bakker’s Balance Model, and AVG. Bakker’s ‘Balance model’ (Bakker and Licht, 1986) provides both an analysis of the reading acquisition process and a consequently structured intervention model for the remediation of reading impairments. The model classifies dyslexic readers into different types, according to the persistent over-reliance on specific hemispheric reading strategies: L-type dyslexic readers predominantly use left hemispheric strategies (i.e., linguistic anticipation) and are characterized by relatively fast but inaccurate reading; P-types use right hemispheric strategies (i.e., perceptual analysis) and are characterized by relatively slow but accurate reading; M (mixed)-types strive to use both kinds of strategies but do so inefficiently, therefore their reading is both slow and inaccurate. A theory-based treatment program was derived from Bakker’s model, aimed at drawing the less involved hemisphere into the process of reading by manipulating sensory stimulation, stimulus characteristics, and tasks. Hemispheric-specific stimulation is carried out by tachistoscopic presentation of words to a visual hemifield in order to selectively stimulate right-hemisphere perceptual analysis or left-hemisphere linguistic anticipation. A computerized program is used, in which the word is flashed and presented for less than 350 ms (too short to allow the eyes to move and align to the word, which would imply bringing the stimulus to the central visual field and losing the hemisphere-specific nature of the stimulation) only if the child clicks on the mouse at the exact moment a dot is crossing a central target (control of eye position and fixation). *Ad hoc* created lists of stimuli and tasks provide stimulation of linguistic anticipation-based (left-hemisphere) vs. perceptual-based (right-hemisphere) reading strategies (see, Lorusso et al., 2006). AVG training was designed to overcome attentional dysfunctions characterizing dyslexic individuals and affecting both visual-spatial attention and serial visual search abilities fundamental for the perception of stimuli during reading (Franceschini et al., 2012, 2013). AVGs feature some qualitative characteristics – such as high speed, a high degree of perceptual and motor load, the presence of several simultaneously moving elements close to each other (crowding), unpredictability of movement, and an emphasis on peripheral processing – which specifically improve visuo-spatial attention abilities and have effects on the rapid focusing and orienting of attention during visual tasks. Based on these evidences, AVG training involves participants with DD in a video-game training, specifically aimed at improving visuo-spatial attentional abilities. A commercial WiiTM video game from UbisoftTM (suitable for children age 7 and older by the Pan European Game Information) called “Rayman Raving Rabbids” is used. Only some of the games included in the DVD have been selected and used, since they possessed all the crucial characteristics needed to be classified as ‘Action Video Games’ (the other games, by contrast, were classified as Non-Action Video Games and used as control tasks in validation studies). AVG training was found to be effective in improving reading in both Italian-speaking (Franceschini et al., 2013; Gori et al., 2016) and English-speaking children with DD (Franceschini et al., 2017). However, no difference between reading improvements after AVG training and the spontaneous development of reading abilities – as measured in a no-treatment control group – has been reported in a Polish population (Łuniewska et al., 2018).

### Procedure

The two experimental groups (RRT and VHSS-AVG) took part in a training program for a total of 13 h over 9 days (two 45-min training sessions per day). The training sessions occurred three times per week for 3 weeks. Such short and intensive intervention model was designed to answer the need for an effective intervention that requires a limited investment of time for both the institution and the families involved. The two methodologies proposed have been previously shown to be effective when applied in relatively short training programs (Lorusso et al., 2006; Franceschini et al., 2013; Bonacina et al., 2015). The sessions were conducted by psychologists expert in the use of each methodology, who were neutral with respect to the hypothesis of the study. The meetings took place in a quiet room of the neuropsychiatry unit of the host institution.

A test-training-retest experimental design was carried out. Before and after intervention, a series of tests were administered by a professional psychologist specialized in cognitive assessment.

### Neuropsychological Measures

Besides reading abilities, a set of neuropsychological functions involved in or related to the process of reading were assessed, in order to measure their degree of involvement in the effectiveness of interventions. Specifically, participants were administered the batteries of tests listed below. Information about medical history, diagnosis, and intellectual functioning measures was collected from each participant’s medical records.

#### Reading

The ability to read aloud text and lists of words and pseudo-words was assessed using the following Italian standardized tests: ‘New MT reading tests for junior high-school’ (Nuove prove di lettura MT per la scuola media inferiore) (Cornoldi and Colpo, 1995), which provides accuracy and speed scores in reading aloud age-normed texts; ‘Assessment battery for Developmental Reading and Spelling Disorders-2’ (Batteria per la valutazione della dislessia e disortografia evolutiva – DDE-2) (Sartori and Job, 2007), in which speed and accuracy scores were computed for word reading (4 lists of 28 words each with different concreteness and frequency of use) and pseudo-word reading (2 lists of 16 pseudo-words each with different lengths). A second test of word and pseudo-word reading, ‘Word and pseudo-word reading test’ (WPRT, Prova di lettura di parole e non parole) (Zoccolotti et al., 2005), was added, in order to have a more accurate control of all psycholinguistic variables: speed and accuracy scores were thus computed for 4 lists of 30 words varying according to length – short vs. long – and frequency of use – low vs. high frequency and 2 lists of 30 pseudo-words varying for length – short vs. long.

#### Lexical Access

Rapid automatized naming ability was assessed using the “Rapid Automatized Naming test (RAN) – Colors” test (Test di denominazione rapida – Colori) (De Luca et al., 2005); in this test, two matrices (10 rows of 5 stimuli each) of 1 × 1 cm colored squares (i.e., black, blue: RGB 51-102-255, red: RGB 221-8-6, yellow: RGB 252-243-5, and green: RGB 31-183-20) are presented. The child is requested to name sequentially each visual stimulus in the matrix as quickly and as accurately as possible. Naming speed (expressed in seconds) and naming accuracy (expressed in number of naming errors) are recorded. Scores are reported as *Z*-scores with respect to age means.

#### Phonological Awareness

Two tasks, namely phonemic blending and phonemic elision (Cossu et al., 1988), were applied to assess phonological awareness skills. Specifically, phonemic elision assesses the ability to recognize and isolate the phonemic constituents of 20 words. The child is asked to omit the first two phonemes of the word pronounced by the examiner and to say aloud the resulting pseudo-word. Phoneme blending assesses the ability to derive a phonemic pattern from distinct phonemic units. The examiner presents 20 words, pronouncing them letter by letter. Then, the child is asked to mentally merge them and pronounce the resulting word. For both tasks, the scores are expressed in total number of errors. Only age means (expressed in raw scores) are provided. To address the mechanisms inducing reading improvements after RRT, several additional tests were administered to RRT group only, measuring the abilities specifically involved in the training. Unfortunately, two participants could not take part in this additional assessment, and other two participants could not complete two of the tasks, namely length and rhythm discrimination. Therefore, complete data are available for eight participants of the RRT group. These additional tests are listed below.

#### Auditory Attention

Auditory selective attention was assessed using the “Selective Auditory Attention Test” from “NEPSY-II” (Korkman et al., 2007). In this task, the child listens to a pre-recorded list of words and touches the appropriate circle in the stimulus book when he or she hears a target word. This subtest comprises two parts, namely “Auditory Attention,” assessing selective auditory attention and the ability to sustain it (vigilance), and “Response Set,” assessing the ability to shift and maintain a new and complex set, involving both inhibition of previously learned responses and correctly responding to matching or mismatching stimuli. The participant listens to a pre-recorded list of words and is instructed to touch the appropriate circle in the stimulus book when hearing a target word. Points are scored only if the child responds correctly within 2 s from the presentation of the target word. Standard scores are provided for the sum of “Auditory Attention” and “Response Set.”

#### Rhythm Perception and Reproduction

Rhythm and sound length discrimination abilities were assessed using the Rhythm Task from the “Q1 VATA Assessment Battery for Cross-domain Learning Abilities” (Batteria per la valutazione delle abilità trasversali all’apprendimento, Q1 VATA) (De Beni et al., 2006), a battery of tests aimed at measuring the abilities mainly involved in school learning processes (i.e., reading comprehension, meta-comprehension, listening comprehension, writing, study skills, reasoning, numeracy, motor coordination, rhythm). The “Rhythm Task” comprises two subtests, namely “Duration” and “Rhythm.” In the “Duration” subtest, sequences of tones are presented, and the participant is asked to identify the tone differing from the others in duration. Each sequence comprises 4–5 tones. In the “Rhythm” subtest, sequences of 6–10 tones are presented in pairs, which can be either identical or different in rhythmic structure. The participant is asked to judge whether the two sequences of each pair are the same or different. Performance in both subtests is expressed as a percentage of correct responses on the total number of trials. Rhythm reproduction ability was measured using the “Rhythm reproduction task” (Stambak, 1951), which consists in reproducing a set of rhythmic patterns of increasing complexity. More precisely, the participant is instructed to reproduce a sequence of 3–8 beats by tapping a pencil on the desk, as demonstrated by the examiner. In the second part of the test, the child is instructed reproduce similar sequences based on a graphic representation of the rhythmic sequences (i.e., squares separated by short or long spaces, representing short or long pauses between the beats). The score expresses the number of incorrectly reproduced sequences (number of errors).

## Results

### Data Analysis

Data were analyzed according to the following steps. First of all, all the pre-test measures of reading ability were compared in the two groups with Mann–Whitney *U* tests for independent samples, to check for possible, casual initial group differences to be taken into account in the following analyses. Subsequently, improvement of reading measures was compared between the two groups. In order to express reading efficiency, global measures of reading performance were computed from the various measures. First of all, mean scores for Reading speed and Reading accuracy were calculated (computed as mean *z*-scores for speed and for accuracy, respectively, on tests of text reading, of word and pseudo-word reading from the DDE battery, and on the various lists of the WPRT). Furthermore, in order to distinguish among the different types of decoding processes elicited by the different types of stimuli, separate global scores were computed for Text reading, Word reading, and Pseudo-word reading, by averaging speed and accuracy *z*-scores for each task. Finally, global Phonological awareness measures were computed as the mean of raw scores (errors) from phonemic blending and elision tasks, to express phonological awareness levels. A first general analysis was conducted using Mann–Whitney *U* tests for independent samples and comparing difference-scores (i.e., post-test minus pre-test *z*-scores) in the two groups. Moreover, the degree of improvement was analyzed by means of Wilcoxon single sample signed-rank tests on pre- and post-test global scores, in each group. In both cases, whenever significant main effects were found, the global score was further analyzed in terms of its constituent tests in order to better describe the nature of the difference or the exact source of improvement. Since all the global scores (or difference-global scores) were computed from the same set of three tests, namely text/word/pseudo-word reading, alpha was divided by three and set at 0.017. Finally, Spearman’s correlations were computed between observed improvements in reading and other reading-related neuropsychological variables. *Post hoc* non-parametric correlations were computed on the component subtests for the variables that turned out to be significantly correlated, in order to better highlight the nature of the association. In order to avoid spurious effects, a cut-off was applied on correlation coefficients, so that only large correlations, conventionally identified with coefficients above 0.50, in addition to *p*-values, were considered significant.

### Effects of the Interventions on Reading and Phonological Awareness

**FIGURE 1:**
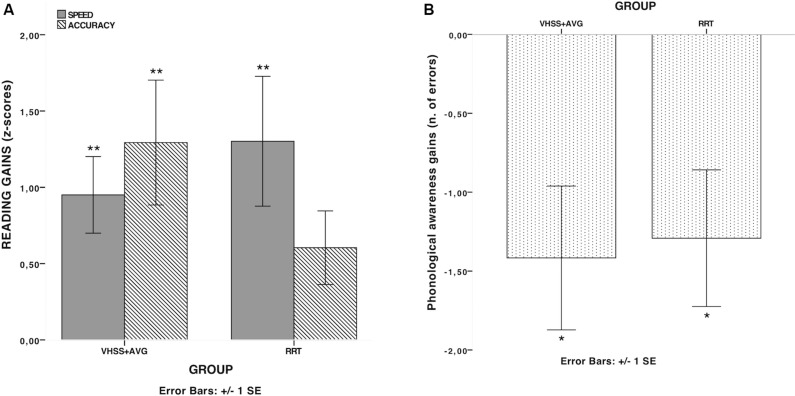
*D*-scores (differences between pre- and post-training scores) for global Reading Accuracy, global Reading Speed **(A)** and global Phonological Awareness **(B)** in the two groups. *p < 0.017; **p < 0.01.

**FIGURE 2:**
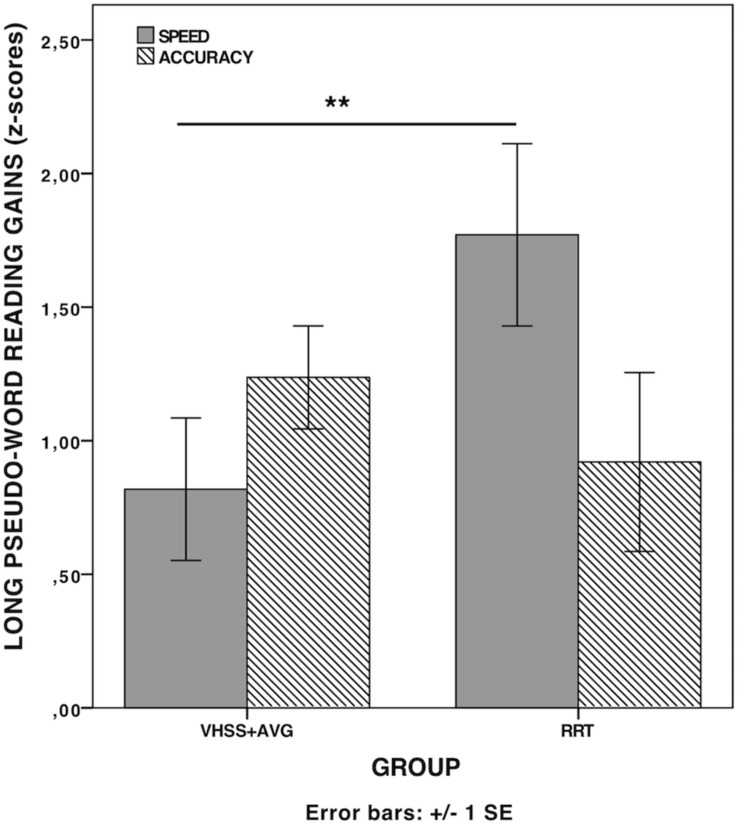
Comparison of mean *D*-scores between long pseudo-word accuracy and speed scores in the WPRT test. **p < 0.01.

**FIGURE 3:**
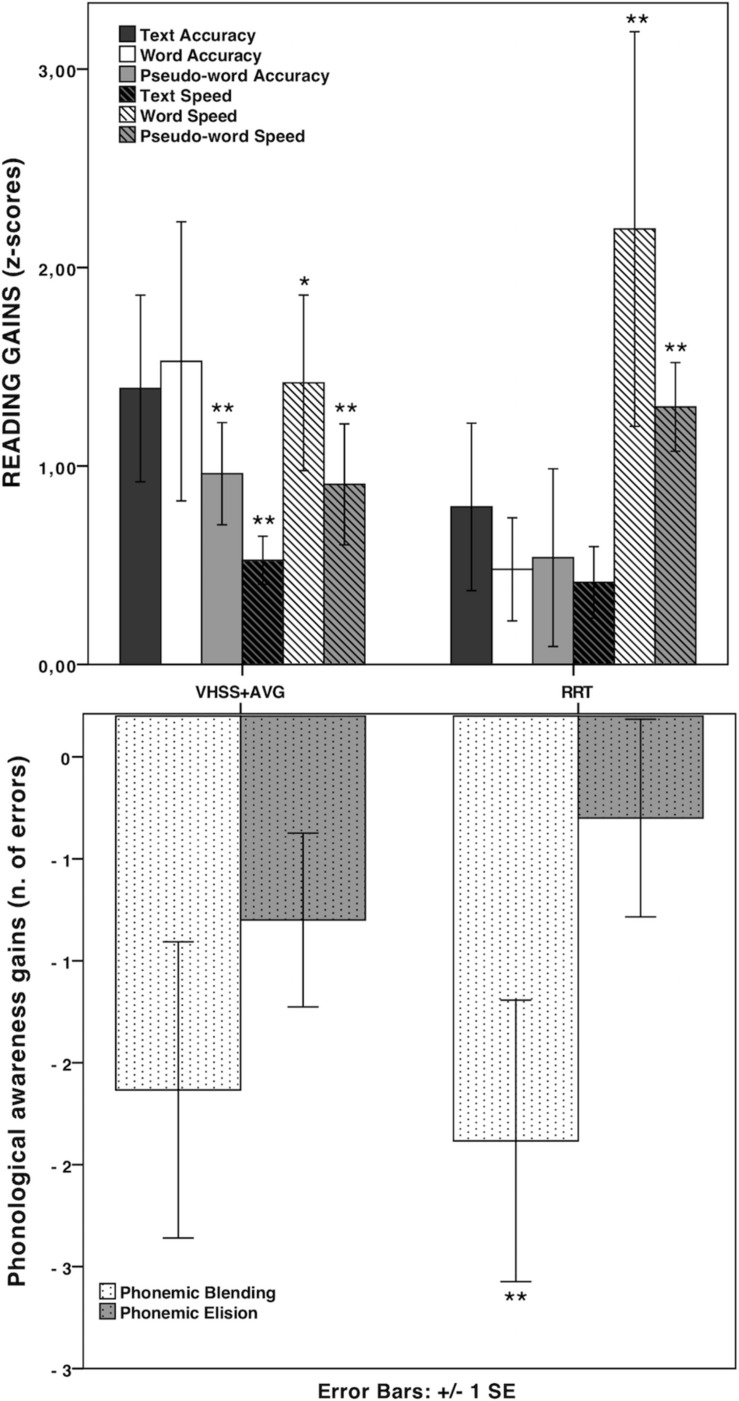
Mean *D*-scores of reading subtests (text reading and DDE word and pseudoword reading test) and phonological awareness tests in the two groups. *p < 0.017; **p < 0.01.

None of the tests (Mann–Whitney’s *U*) on pre-treatment scores showed significant differences between the two groups (all *p*s > 0.10), thus ensuring that the two groups were comparable at pre-test. The results of pre/post-test comparisons (Wilcoxon signed-rank test) for global reading and phonological scores in the two groups are reported in Table 2. A significant improvement in reading speed and phonological measures occurred, thus supporting the notion that both interventions were effective in increasing children’s reading speed and phonological skills (see Figure 1). Global reading accuracy was found to be significantly improved in the VHSS-AVG group only. However, no significant differences were found in any Difference-scores (*D*-scores: Post-test minus pre-test) between the two groups. A further hypothesis concerned the possibility of specific effects of the two interventions on different reading parameters (speed and accuracy) and/or on different subtypes of stimuli (words and pseudo-words, also distinguishing according to stimulus characteristics such as length and frequency). Since no full factorial design was possible in this case (pseudo-words varying according to length but not frequency), a series of analyses were conducted by taking into account words and pseudo-words separately and considering the specific scores on the WPRT, providing distinct lists according to the relevant lexical variables. Mean Difference-scores between accuracy and speed scores in the various lists were computed and compared between the two groups. A significant difference emerged between the RRT group and the VHSS-AVG group with Mann-Whitney *U*-test for long pseudo-word reading speed (*U* = 31.0; *p* = 0.018, *d* = 1.10) (see Figure 2). As anticipated, pre-planned analyses were conducted on component tests of the global scores yielding significant differences. These analyses are reported in Table 3. Mean *D*-scores for the two groups are reported in Figure 3. The tendency for RRT to induce greater effects on speed and for VHSS + AVG to have more significant effects on accuracy was observed throughout most tests, although the comparison between *D*-scores in the two groups never approached significance (see Table 3).

**TABLE 2:** Mean scores (SD in parentheses) and results of non-parametric comparisons of Pre–Post-test Global scores in the two groups, and of between-group comparisons on Difference Global scores (*D*-scores).

		VHSS-AVG *N* = 12	RRT *N* = 12	Pre–post within-group comparison: Wilcoxon signed-rank test z-scores, *p, d*		*D*-scores between-group comparison
		Mean (*SD*)	Mean (*SD*)	VHSS + AVG	RRT	Mann–Whitney *U*-test, *p, d*
Global Reading Speed^a^	PRE	−2.440(2.21)	−2.508(2.28)	**2.982, 0.003, 1.540**	**3.059, 0.002, 1.600**	62.00, 0.564, 0.235
	POST	−1.489(2.13)	−1.206(0.993)			
Global Reading Accuracy^b^	PRE	−2.675(2.51)	−2.301(1.852)	**2.981, 0.003, 1.540**	2.040, 0.041, 0.915	57.00, 0.386, 0.357
	POST	−1.382(1.29)	−1.700(1.702)			
Global Phonological awareness^c^	PRE	3.042 (2.13)	3.333 (1.958)	**–2.466, 0.014, 1.164**	**–2.380, 0.017, 1.112**	69.5, 0.887, 0.058
	POST	1.625 (1.07)	2.042 (1.514)			

*Significant differences in bold. α = 0.05/3 = 0.017. Effect sizes are reported as Cohen’s *d*. ^*a*^Mean *z*-scores of text, word and pseudo-words (from DDE battery) reading speed. ^*b*^Mean *z*-scores of text, word and pseudo-words (from DDE battery) reading accuracy. ^c^Mean raw scores (n. of errors) in phonemic blending and elision.*

**TABLE 3:** *Post hoc* tests on component subtests: results of non-parametric comparisons of *D*-scores (text reading and DDE word and pseudoword reading test) within the two groups, and of between-group comparisons.

	*D*-scores expressed in raw scores^a^		*D*-scores expressed in *z*-scores		Pre–post-test comparison (*D*-scores, against the hypothesis of null gains)		Group comparison on *D*- Scores
	VHSS-AVG *N* = 12 Mean (*SD*)	RRT *N* = 12 Mean (*SD*)	VHSS-AVG N = 12 Mean (*SD*)	RRT *N* = 12 Mean (*SD*)	Wilcoxon signed-rank test z-scores, *p, d*		
					VHSS-AVG	RTT	Mann–Whitney *U*-test, *p, d*
Text reading Accuracy	−	−	1.391 (1.63)	0.794 (1.46)	2.312, 0.021, 1.071	1.923, 0.055, 0.852	60.5, 0.514, 0.272
Word reading Accuracy	−	−	1.527 (2.43)	0.479 (0.90)	1.961, 0.05, 0.873	1.804, 0.071, 0.791	49.0, 0.198, 0.563
Pseudo-word reading Accuracy	−	−	0.961 (0.89)	0.538 (1.55)	**2.936, 0.003, 1.496**	1.255, 0.209, 0.529	63.0, 0.630, 0.213
Text reading Speed	0.338 (0.27)	0.338 (0.26)	0.525 (0.42)	0.414 (0.62)	**3.061, 0.002, 1.601**	2.118, 0.034, 0.958	69.0, 0.887, 0.070
Word reading Speed	0.400 (0.31)	0.455 (0.33)	1.419 (1.53)	2.194 (3.44)	**2.510, 0.012, 1.192**	**3.059, 0.002, 1.597**	58.0, 0.443, 0.335
Pseudo-word reading Speed	0.185 (0.12)	0.308 (0.23)	0.907 (1.05)	1.298 (0.77)	**2.746, 0.006, 1.352**	**3.061, 0.002, 1.601**	46.0, 0.143, 0.643
Phonemic Blending	−1.83(2.52)	−2.08(2.39)	−	−	2.082, 0.037, 0.939	**2.671, 0.008, 1.300**	70.0, 0.932, 0.048
Phonemic Elision	−1.00(1.48)	−0.50(1.68)	−	−	1.997, 0.046, 0.891	0.862, 0.389, 0.370	55.5, 0.347, 0.406

*Significant differences in bold. α = 0.05/3 = 0.017. Effect sizes are reported as Cohen’s *d*. ^*a*^Raw scores are reported for reading speed (expressed in syllable per second) and for phonemic blending and elision tasks (expressed in number of errors).*

### Spearman’s Correlation Coefficients

A correlation between Phonological awareness improvement (as measured by the phonemic blending task) and Text reading performance improvement (computed as mean *z*-scores of text reading speed and accuracy gains) was found in the RRT group (ρ = –0.694, *p* = 0.012; the correlation is negative as phonological awareness abilities are expressed as number of errors, whereas reading performance as mean *z*-scores). At a *post hoc* analysis, it could be observed that correlations of text reading improvements concerned both speed and (to a lesser degree) accuracy (ρ = –0.756, *p* = 0.004 for speed; ρ = –0.599, *p* = 0.039 for accuracy). In the VHSS + AVG group, improvements in text reading correlated with improvement in RAN speed (ρ = 0.585, *p* = 0.048). This correlation emerged mainly from text reading speed (ρ = 0.630, *p* = 0.028) and, to a lesser degree, from accuracy (ρ = 0.573, *p* = 0.051). As to the initial variables that were found to correlate with reading improvement in the two groups, initial levels of Phonemic blending (errors) correlate with improvement in Text Reading (ρ = 0.723, *p* = 0.008) in the RRT group, while initial RAN speed negatively correlates with improvement in Text reading (ρ = –0.775, *p* = 0.005) in the VHSS-AVG group. Finally, no correlations between reading improvements and either IQ measures or age have been found.

### Effects of RRT on Rhythm Perception/Reproduction and Auditory Attention

**FIGURE 4:**
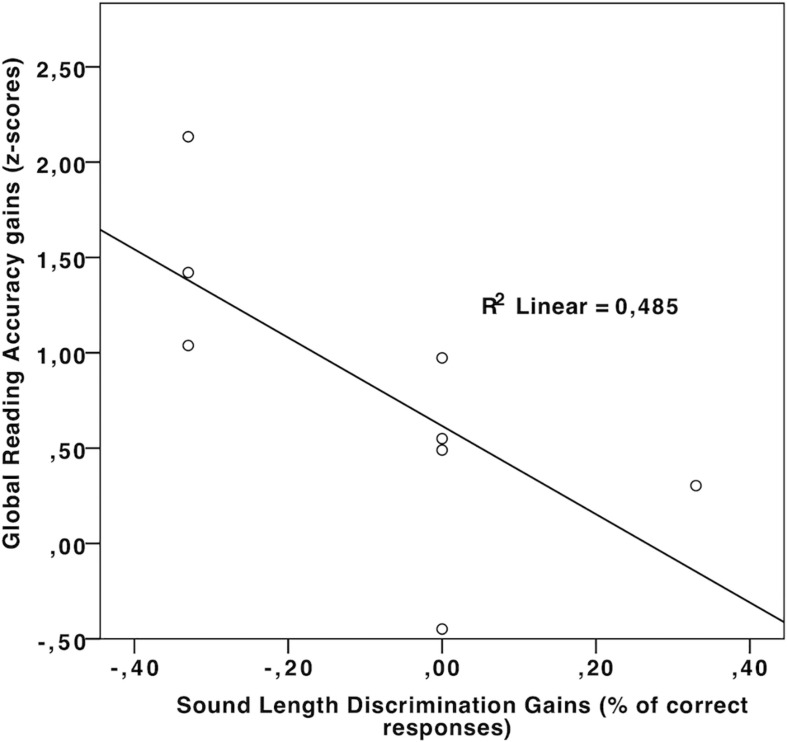
Scatterplot illustrating the correlation between improvement in Sound Length discrimination and improvement in reading accuracy, in the RRT group.

**FIGURE 5:**
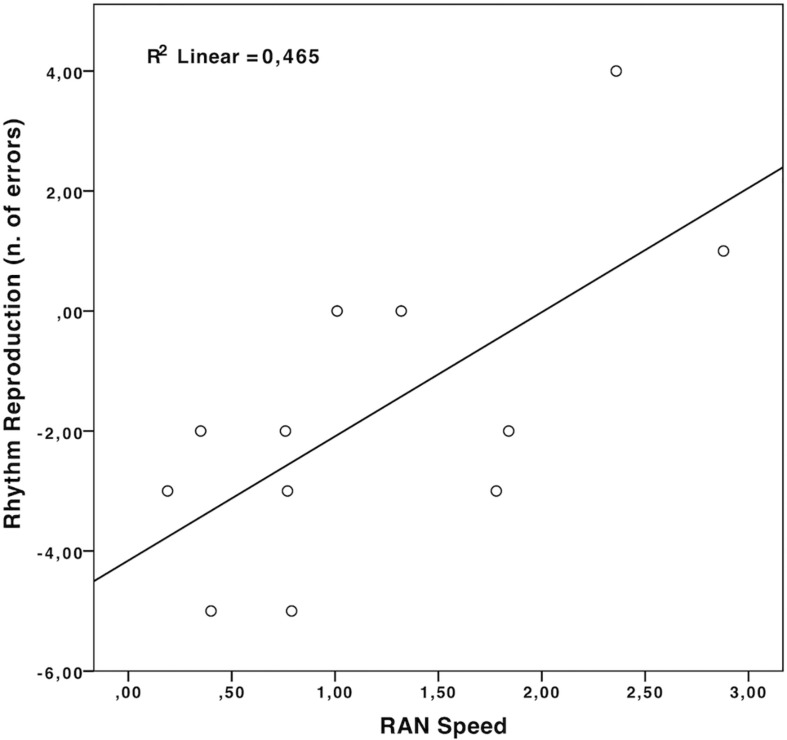
Scatterplot illustrating the correlation between improvement in Rhythm Reproduction scores (Stambak test) and improvement in RAN speed scores, in the RRT group.

Comparing pre- and post-test performance with Wilcoxon signed-rank test (against the hypothesis of null gains, see Table 4), observed improvements in rhythm perception and auditory attention in the RRT group did not reach statistical significance after correction for multiple comparisons (four tests,α = 0.012). These results suggest that RRT affects both reading abilities and rhythmic skills, although its effects on reading are more evident than those on rhythmic and auditory abilities. Nonetheless, correlations showed an interesting picture (see Table 5), with significant negative correlations emerging between improvements in Sound length discrimination and in both Reading Accuracy and RAN accuracy (see Figure 4). A similar negative correlation was found between improvement in Rhythm Reproduction (Stambak test) and improvement in RAN speed (see Figure 5).

**TABLE 4:** Effects on rhythm perception and auditory attention for the RRT group: mean scores (SD in parentheses) and results of Wilcoxon signed-rank test for sound length discrimination, rhythm discrimination, rhythm reproduction and selective auditory attention abilities.

		Mean (*SD*)	Wilcoxon signed-rank test on *D*-scores z-scores, *p*
Length discrimination^a^	PRE	0.577 (0.15)	–1,0.317, 0.516
	POST	0.495 (0.17)	
Rhythm discrimination^a^	PRE	0.831 (0.25)	0.378, 0.705, 0.189
	POST	0.832 (0.35)	
Rhythm reproduction^b^	PRE	5.92 (2.75)	1.897, 0.058, 1.077
	POST	4.25 (2.67)	
Selective auditory attention^c^	PRE	99.08 (17.75)	–1.379, 0.168, 0.735
	POST	103.5 (13.69)	

*α = 0.05/4 = 0.012. Effect sizes are reported as Cohen’s *d.*^*a*^Scores are expressed as percent correct responses in the sound length and rhythm discrimination tasks. ^*b*^Raw scores (n. of errors) in the Stambak rhythm reproduction task. ^*c*^Standard scores in the selective auditory attention task of the Nepsy-II.*

**TABLE 5:** Spearman correlations between improvements observed in rhythmic/auditory attention abilities and in reading/reading related skills, in the RRT group.

RRT GROUP		D_Length Discrim	D_Rhythm Discrim.	D_Rhythm Reprod.	D_Select. Audatt
Spearman	D_global Read.Speed N	0.548	0.485	–0.132	–0.084
Rho		0.160	0.223	0.683	0.795
		8	8	12	12
	D_global Read.Accuracy N	**–0.848**	–0.281	0.014	–0.014
		**0.008**	0.500	0.965	0.966
		**8**	8	12	12
	D_global Phon.Aware. N	–0.072	–0.128	–0.065	–0.057
		0.865	0.762	0.841	0.861
		8	8	12	12
	D_RAN Col. Accuracy N	**–0.782**	–0.064	–0.114	–0.175
		**0.022**	0.881	0.724	0.587
		**8**	8	12	12
	D_RAN Col. Speed N	0.156	0.192	**0.605**	–0.42
		0.711	0.650	**0.037**	0.175
		8	8	**12**	12

*Legend: Read., reading; Phon.Aware., Phonological awareness; Col., color; Reprod, reproduction; Audatt, auditory attention; Select., selective; Discrim., discrimination. Significant correlations in bold.*

## Discussion

The purpose of the present study was to compare the effectiveness of the RRT, an intervention method for DD in which reading exercises are integrated with rhythm processing, with that of an intervention resulting from the combination of two already validated treatments for DD (VHSS according to Bakker’s Balance Model and AVG). Results showed that both interventions were significantly effective in improving reading speed and reading accuracy in a group of students with DD. A slightly larger improvement in reading speed due to the participation in the RRT and larger improvement in reading accuracy (especially in text reading) after the VHSS-AVG intervention was observed. However, differences between groups in global outcomes were statistically non-significant. Significantly greater improvements in pseudoword reading speed were induced by RRT, therefore suggesting that grapheme–phoneme conversion mechanisms are better supported by improved attending to the rhythmic structures underlying phonological structure of language. According to our initial hypothesis, RRT could indeed help the child to segment the hierarchical acoustic rhythm structures in language that map to phonological units, by stressing syllables and onset-rimes.

Both interventions featured combined trainings, integrating reading-based exercises with the training of more low-level functions, respectively auditory-perceptual (for RRT) and visual-attentional functions (VHSS-AVG). We suggest that this very combination was the key to the effectiveness of both interventions.

Based on observed correlations, it could be hypothesized that RRT acts on phonological processing, but also forces the child to adapt to an external rhythm imposed by the software, thus boosting increase in processing speed rather than accuracy of performance. The larger effects on accuracy observed in the VHSS-AVG group, by contrast, are likely to depend on the VHSS component, which was shown in previous studies to be particularly effective in enhancing reading accuracy (Lorusso et al., 2004, 2006, 2011), whereas AVG trainings appear to be especially effective in boosting speed (Franceschini et al., 2013). The analysis of correlations suggests, though, that improvements in reading are elicited by different mechanisms and processes in the two groups.

### Phonological Awareness and Rapid Naming Distinctly Related to Reading Improvement in RRT and VHSS-AVG

Concerning phonological awareness, which is usually assumed to be particularly linked to sublexical reading abilities (Harm and Seidenberg, 1999; Shapiro et al., 2013), significant improvements were found in both groups to a similar extent. In the RRT group, phonological gains (especially in phonemic blending, which is indeed most strictly related to decoding processes) were also related to text reading performance improvements. Separate correlations for speed and accuracy of text reading showed that reading speed improvement is especially related to phonological awareness gains (specifically in the phonemic blending task). It should be considered that text reading tests require reading of both high and low frequency words, thus involving the activation of mechanisms usually linked to both word and non-word reading. Therefore, in the RRT group, phonological awareness may have supported the improvement of text decoding speed. As far as the VHSS-AVG group is concerned, by contrast, improvement in text reading correlates with improvement in speed of RAN: this correlation depends to a very similar extent on text reading speed and accuracy. Since RAN underlies orthographic processing, namely, when groups of letters or entire words are processed as whole-unit lexical access stimuli rather than as a sequence of grapheme–phoneme correspondences (e.g., Georgiou et al., 2009), this pattern of correlations suggests that different mechanisms may have been empowered by the two different trainings. On the other hand, these results are consistent with findings showing that both RAN and phonological ability contribute independently to the prediction of reading ability (accuracy and speed) in the Italian language, which has a shallow orthography (Di Filippo et al., 2005). This is a novel finding with respect to the VHSS-AVG treatment. Indeed, in previous studies VHSS had been found to improve verbal memory along with phonological awareness, as well as selective visual-spatial attention (Facoetti et al., 2003; Lorusso et al., 2006, 2011); AVG had been found to improve both phonological working memory (including phonological awareness measures) and visual-spatial attention (Franceschini et al., 2013, 2017). In both cases, the improvements in reading and in the above-mentioned abilities were correlated, suggesting that a causal relationship between the latter and the former is possible (even if not demonstrated). The reduced sample size moreover calls for great caution in generalizing such results. Since previous studies on VHSS and AVG did not include RAN measures, the (possible) role of RAN in reading improvement could be either a product of the (new) combination of the two approaches or an additional factor that has been highlighted in the present study for the first time. Similarly, the absence of a correlation with phonological awareness could be an effect of the combination of the two treatments. Such hypotheses need to be confirmed with larger samples.

### Additional Results

Finally, the additional tests that were designed to detect changes in the specific abilities stimulated by the RRT program allowed investigators to get interesting results. In particular, improvement in discrimination of sound length highly correlates with improvements in reading accuracy, but in a negative direction. If considering that sound length discrimination shows no improvement (rather, a non-significant decrease) from pre-test to post-test (the only auditory test that shows improvement is the Stambak rhythm reproduction test with increased percentages of correct responses), this suggests that improvement in reading accuracy is observed in spite of decrease in auditory discrimination. In order to better interpret this puzzling result, initial variables have been taken into account as determiners of improvement: correlations show that better scores in sound length discrimination prior to treatment are positive predictors of improvement in Word and Pseudo-Word reading accuracy (as found, respectively, in the DDE-2 and in the WPRT, ρ = 0.756, *p* = 0.030 in both cases). This means that children who were more sensitive to sound length characteristics at the beginning of treatment were more prone to improve in reading accuracy. At the same time, though, children with the highest performance in sound length discrimination at pre-test were also those who improved less (possibly because of a ceiling effect) from pre-test to post-test in the same task. It can thus be hypothesized that children with better starting sensitivity to sound characteristics could be advantaged because a higher level in this ability might create better conditions to improve reading accuracy, in spite of the treatment producing limited improvements (or even slight worsening) in sound analysis. In other words, even when auditory analysis does not *improve along* with decoding improvement, it may provide initial conditions favoring the effectiveness of the training. By contrast, phonological awareness in the RRT group and rapid lexical access in the VHSS-AVG group appear to be the neuropsychological mechanisms linked to change and improvement. By the way, lexical access is crucial to both word and text reading, and this link to RAN improvement, together with a specific emphasis on word retrieval strategies in the VHSS + AVG training group, could explain the tendency for this program to improve text and word reading more than pseudo-word reading. The correlation between improvements in reading accuracy and improvements in phonological awareness found in the RRT group supports the hypothesis that the specific mechanism of action of RRT have to be found within the temporal sampling framework proposed by Goswami (2011) and Goswami et al. (2014). According to this hypothesis, stressing the metrical structure underlying language would induce a fine-grained analysis of the temporal constituents of the auditory signal, thus supporting the mapping of phonological units, and resulting in consistent phonological awareness and reading improvements. It can be concluded that a simple rhythmic reading approach, possibly through enhanced phonological awareness, produces a general reading improvement, consistently with previous research on the crucial role of rhythmic auditory skills in reading abilities (e.g., Huss et al., 2011).

### Limitations and Future Studies

The lack of experimental measures specifically tapping the auditory skills that are hypothesized to be mediating the change in reading ability according to the temporal sampling framework (existing tests had been chosen instead, in order to ensure standardized and validated measures, under the expectation that improvements in auditory processing would have been generalized to a broader set of tasks) is acknowledged as a limitation of the study.

A further limitation of the study is the relatively modest number of participants in the two groups; however, the accurate matching of the children for all relevant variables, the use of convergent measures for reading skills and the relatively clear-cut effects that emerged from the analyses suggest that the results can be considered sufficiently reliable. Indeed, it had been shown in a previous study by Lorusso et al. (2006) that VHSS produced an increase in text reading speed of 0.56 syllables per second over a treatment period of 4 months (32 bi-weekly sessions of 45 min each, amounting to about 24 h total time), whereas a control treatment produced an increase of only 0.11 syll/s over the same time. In the present study, increase in syll/s was 0.34 for both groups, over 3 weeks treatment (18 sessions three times per week, with 2 45-min sessions each time, amounting to about 13 h total time). Compared with published data (Tressoldi et al., 2001) on spontaneous changes in reading speed in Italian children with dyslexia (about 0.3 syll/s. in a year) and with typical development (about 0.5 syll/s in a year), the present data confirm the effectiveness of VHSS treatment, also combined with AVG training, and show that RRT has comparable effects on reading speed, both treatments producing in 3 weeks an increase similar to that observed in a whole year without treatment.

Furthermore, we exclude the potential influence of test-retest effects, in spite of the repetition of the same battery of tests 4 weeks apart. A previous study on the efficacy of RRT in Italian children (Bonacina et al., 2015), employing the same reading tests as the present one (i.e., ‘New MT reading tests for junior high-school’ for text reading, and ‘Word and pseudo-word reading test’ for word and non-word reading) which have been re-administered after 4 weeks, included a no-treatment condition to control for potential confounding factors, such as spontaneous reading development and/or test-retest effects. More precisely, no substantial improvement of *z*-scores was reported: the pre-post-test difference in the control group was 0.23 (*SD* = 1.04) for word reading accuracy, 0.20 (*SD* = 0.55) for word reading speed, 0.13 (*SD* = 1.15) for non-word reading accuracy, 0.02 (*SD* = 0.37) for non-word reading speed, –0.13 (*SD* = 1.97) for text reading accuracy and 0.17 (*SD* = 1.97) for text reading speed. Therefore, the improvements after both RRT and VHSS-AVG measured in the present study should be considered sufficiently reliable in reflecting an actual change in ability.

Nonetheless, generalization should be cautious, especially regarding correlations, and larger studies are being planned for replication. Future studies should also allow to precisely document changes in specific auditory processing measures relating to onset-rime discrimination and temporal sampling processes (Goswami, 2011; Huss et al., 2011; Goswami et al., 2014). It will also be important to collect further data on the generalization and consolidation of the improvements after intervention.

## Conclusion

In conclusion, RRT appears to be an effective alternative to other intervention methods for DD. In particular, RRT seems to be especially useful for improving reading speed, whereas VHSS-AVG produces larger effects on accuracy. Based on the correlations found between improvement and initial profiles (to be confirmed with larger samples), it could further be suggested that RRT is most likely to be effective when phonological awareness needs to be stimulated, but auditory analysis is of sufficiently developed, whereas VHSS-AVG seems to be of greater advantage to children with initial impairments in rapid naming. In comparison with traditional treatments, RRT is a very easy-to-use and adaptable training method, which does not require a specific administration setting or demanding training for its application. Additionally, RRT seems to have the advantage of being more inclusive as compared to other known music-based trainings (see Gordon et al., 2015) because it does not require a trainer with particular musical expertise or even a specific facility to practice in. Moreover, it does not exclude children with scarce musical aptitude or interest, as a specific music training could do. Finally, the use of rhythm and music provides an enjoyable and pleasant environment for children who participate in reading training, which contributes to improving their involvement and motivation. The children who took part in the present study generally expressed positive feedbacks about both types of trainings and kept a high level of engagement and motivation throughout the whole duration of the programs, in spite of their intensive schedule. This allowed to reach effects comparable to more traditional intervention schedules (typically, two sessions per week for 10–15 weeks) in a much shorter period and with less time devoted to the training for both the children and the therapists.

## Data Availability Statement

The datasets generated for this study are available on request to the corresponding author.

## Ethics Statement

The studies involving human participants were reviewed and approved by Ethics Committee of the Catholic University of the Sacred Heart, Milan, Italy. Written informed consent to participate in this study was provided by the participants’ legal guardian/next of kin.

## Author Contributions

AC, AA, ML, and SB: conceptualization. AC and ML: methodology and formal analysis. SB and ML: investigation. ML and AC: data curation. AC, ML, and SB: writing—original draft preparation. AC and ML: writing—review and editing. AA, AS, and MM: supervision. AA and MM: project administration. All authors read and agreed to the published version of the manuscript.

## Conflict of Interest

The authors declare that the research was conducted in the absence of any commercial or financial relationships that could be construed as a potential conflict of interest.
